# Task-Dependent Eye-Movement Patterns in Viewing Art

**DOI:** 10.16910/jemr.13.2.12

**Published:** 2020-12-16

**Authors:** Nino Sharvashidze, Alexander C. Schütz

**Affiliations:** Philipps-Universität Marburg, Germany

**Keywords:** art perception, task-dependent viewing, expertise, confidence, viewing strategies, eye movements, scanpath, eye tracking

## Abstract

In art schools and classes for art history students are trained to pay attention to different aspects of an artwork, such as art movement characteristics and painting techniques. Experts are better at processing style and visual features of an artwork than nonprofessionals. Here we tested the hypothesis that experts in art use different, task-dependent viewing strategies than nonprofessionals when analyzing a piece of art. We compared a group of art history students with a group of students with no art education background, while viewing 36 paintings under three discrimination tasks. Participants were asked to determine the art movement, the date and the medium of the paintings. We analyzed behavioral and eye-movement data of 27 participants. Our observers adjusted their viewing strategies according to the task, resulting in longer fixation durations and shorter saccade amplitudes for the medium detection task. We found higher task accuracy and subjective confidence, less congruence and higher dispersion in fixation locations in experts. Expertise also influenced saccade metrics, biasing it towards larger saccade amplitudes, advocating a more holistic scanning strategy of experts in all three tasks.

## Introduction

### Task-driven selection

It is widely agreed that visual attention is on one hand stimulus-driven, induced by the physical properties of an image, referred to as salience, categorized as bottom-up processing. On the other hand, top-down demands like current goals, task, knowledge, expectations and reward also direct our attention (1, 2). Stimulus-driven models of attention claim that where the observer first looks at in a scene is determined by the low-level features of a scene. Strong contrasts in brightness, color and orientation stand out from the rest of the scene and grab the attention of the viewer (3). Bottom-up processing has been shown to drive our attention due to low level feature correlation with object properties ( 4). Generally, bottom-up models have been more successful in explaining fixations in free viewing (5) but they come to their limits when observers start to pursue specific goals (6). Previous research has shown that salience can explain only a small portion of fixations in everyday tasks. During everyday activities like driving (7), making tea ( 8) or sandwiches (9) observers only fixate task-relevant areas and ignore task-irrelevant, although salient areas. Buswell was probably first to notice that, “The mental set obtained by the directions given for looking at a picture obviously influences the characteristics of the perceptual process” (10). However, one of the most famous examples of task-driven visual guidance comes from Yarbus who used paintings to demonstrate the influence of different tasks on the eye movements (11). For instance, if the observer was asked to remember the clothes of depicted people, mainly clothes were attended, whereas if the observer had to estimate the age of people, the most informative areas for this particular task—faces, were fixated. His demonstration has been replicated a few times under more valid conditions with some modifications leading to comparable results (12, 13, 14).

Fixation count and fixation durations, saccade count and saccade amplitudes are useful estimates for the task influence on scene processing (15, 16, 17, 18, 19). Castelhano et al. (20) found that task affected where the observers fixated. Fixations were widely distributed in the memorization task and more focused on search-relevant areas in the search task. They were also able to find differences in the saccade amplitude during the initial viewing of the scene. Dynamics of fixation and spatial fixation density seem to be important factors that are variable as a function of a task (21).

Numerous different tasks have been used to explore task-dependent visual selection while viewing art, including free viewing (22, 23), judging the aesthetic quality (24, 25, 26, 27), paying attention to semantic content (26), memorization (28), categorization and person detection ( 29), movement detection (25). Despite the diversity of tasks, to the best of our knowledge no common art historian tasks have yet been used to examine task-driven viewing patterns while looking at paintings. Conventional art expertise tasks, including accessing the value or the authenticity of the artwork go hand in hand with the recognition of style and art movement, medium and technique, as well as the correct dating of the artworks. Accurate assessment of the art movement requires knowledge about art periods, as well as understanding of characteristic categories of depicted subjects and content and distinctive modes of expression. Date estimation involves some further background information about historical context and the timeline of art history. Defining the physical characteristics of the painting includes determining the materials and tools that artists use to create a work of art. The knowledge required to solve all these tasks are passed on in art schools and classes for art history.

### Expertise

Expertise is broadly defined as constantly superior performance within a specific domain relative to novices (30). Expertise criteria used in different studies in the visual domain include performance efficiency, social recognition, group membership and years of experience ( 31). By trying to summarize what contributes to the expert superior visual performance, Gegenfurtner et al. (31) considered three theories supported by the literature in their meta-analysis (32). The theory of long-term working memory (33) assumes that experts encode and retrieve information more rapidly than novices. According to this theory, experts’ rapid information processing should be reflected in shorter fixation durations. The information-reduction hypothesis (34) focuses on learned selectivity of information processing. It proposes that expertise optimizes the amount of processed information by neglecting task-irrelevant information and focusing on task-relevant information. This results in more fixations on task-relevant areas and fewer on irrelevant areas. The holistic model of image perception (35), proposes the idea that experts are able to extract information from widely distanced and parafoveal regions. This model anticipates larger saccade amplitudes and shorter time to first fixate task-relevant areas in experts.

Where/what does one have to attend in order to extract useful information seems to depend on the scene context and knowledge of the scene statistics. Scene statistics that are learned from experience are linked to full range of selection history effects (36, 37, 38, 39). Experts’ ability to extract task-relevant information is linked to their knowledge of scene context and scene statistics based on past visual experience and declarative knowledge about the paintings. Generic semantic and spatial knowledge about the particular type of scene is described as scene-schema knowledge (40). For example, it includes information about the objects that are likely to be found in this scene category. Scene-schema can be rapidly retrieved and used to limit fixations to scene areas that are likely to contain task-relevant information. This could be information about how paintings are generally organized, about the typical compositional structures and the common subjects that are depicted. Another type of knowledge, task-related knowledge can involve a general “gaze-control policy” or strategy relevant to a given task (40), knowledge about style specific regularities, understanding of how the mixes of pigments and binding media usually look like, how they are applied and where they can be conveniently identified. The same assumption is illustrated in the Leder et al.’s (41) model of aesthetic experience, where past experience with art, for example, exposure to artworks and declarative knowledge can impact the experience on separate levels. On the one hand, previous experience, which also interacts with domain specific expertise affects implicit memory integration, on the other hand, domain specific declarative knowledge influences explicit classification and enables, content vs. style processing. Therefore, in the case of art experts, one encounters the combination of visual experience, due to the exposure to many paintings throughout art classes and a comprehensive declarative knowledge about art.

Augustin and Leder (42) showed that, while non-experts merely use terms familiar to them from everyday experience, experts relate artworks according to the style, art movement or other art-specific aspects. Buswell (10), who was the first to compare fixation patterns of artistically trained and untrained subjects, could not find any systematic differences between the groups. He was, however, quite tentative about the nonexistence of the differences and remained critical of his own methods. Indeed, later research has provided some evidence that experts and non-experts differ in how they look at paintings. Nodine et al. (43) analyzed eye movements of professional and nonprofessional viewers, who judged compositions that differed in balance through manipulation of composition symmetry. They found a more global strategy of scanning an image in experts. The same pattern was found by Zangemeister et al. (28). Experts also tend to make less frequent and shorter fixations on narrative elements (27, 44, 45). Ylitalo et al. (46) found that experts tend to have shorter fixation durations and less variability in fixation durations than novices. Fedorovskaya et al. (47) found less local viewing by experts and less re-fixations on already attended areas. Pihko et al. (27) demonstrated that gaze patterns of non-experts became closer to those of experts after they received additional information about the painting.

To sum up, evidently, diverse tasks have been investigated in the context of visual art, however, previous studies have mainly focused on free viewing and aesthetic judgment tasks. Art-specific tasks have been overlooked. Art historians, experts in conservation and restoration encounter domain-specific tasks on a daily basis. However, we do not know how the specific task demands affect their eye movement patterns and viewing strategies. Eye movement parameters and spatial fixation density seem to vary from task to task, thus we assume that art-specific tasks will also induce variation in fixation dynamics and attended painting areas. Regarding expertise, we assume that experts, having scene-schema knowledge, task-specific and declarative knowledge about the paintings, establish different task-dependent strategies than non-experts. It is likely, that they generally, develop hypotheses regarding a painting and control gaze in order to efficiently solve the problem. For instance, Nodine et al. (43) showed that experts are sensitive to composition and use structure of a painting to allocate their attention to the informative parts of the painting. Bauer and Schwan (48) showed that experts are more effective in searching for content that is helpful for successful meaning-making in the Renaissance portraits. Accordingly, experts seem to be better at looking for specific aspects of an artwork that are relevant for the task.

In this study, we examined experts and non-experts in art by giving them three different art related tasks to solve. Participants were asked to define the style/art movement, estimate the date and discriminate the medium of the paintings. In other words, they were asked to identify how, when and whereby the painting was created. The major disadvantage of stimulus sets used to date is the inclusion of paintings with highly salient areas, including faces and texts. With this in mind, we aimed to create a better-suited set of stimuli omitting highly salient face areas and incorporated texts, which are known to quickly attract gaze (49) and can, in some cases, work as cues for the tasks. We aimed to explore familiarity of paintings, accuracy and confidence ratings of our observers. We expected to see task-driven effects on spatial and temporal characteristics of eye movement behavior.

## Methods

### Participants

It total 29 subjects (age: *M* = 25.06, *SD* = 3.8, range: 19–35 years, 5 male, 3 left-handed) participated in our study. 13 Experts (age: *M* = 25.38, *SD* = 3.25, 2 male) were recruited from art related Marburg University courses including History of Art B.A. (5), History of Art M.A. (6), Concepts of Fine Arts M.A. (1) and Visual Arts, Music and Modern Media B.A. (1). 16 non-experts (age: *M* = 24.81, *SD* = 4.33, 3 male) were students of the following art unrelated university courses: Psychology (6), Political Science (2), Business Administration (1), Sociology (1), Human Medicine (1), German Language and Literature (1), Islamic Studies (1), Educational Science (1), Romance Studies (1) and German as a Foreign Language (1). The data from two non-expert subjects had to be excluded. One due to the technical issue during the experiment (Subject 20), the other due to the poor quality of the eye movement data (Subject 33), resulting in a total sample size of 27 subjects with 13 experts and 14 non-experts. The aforementioned art related university courses offer “Basics of Art History” (History of Art B.A.) or “Propaedeutics of Art” (Visual Art, Music and Modern Media B.A.) as a compulsory class in the first semester. Over the whole course more classes are offered, providing the graduates with thorough knowledge of the important artworks, genres and techniques from late antiquity to the present. All subjects had normal or corrected to normal vision and gave informed consent prior to participation. All of them had normal color vision according to the Ishihara Test (50). They were paid 8€/h and received a book at the end of the experiment. The experiment was in accordance with the principles of the 1964 Declaration of Helsinki and was approved by the ethics committee of the Marburg University, Department of Psychology (proposal 2017-27k).

### Apparatus

The experiment was conducted using the Psychtoolbox (51, 52) in MATLAB (R2016a; The MathWorks, Natick, MA) and stimuli were presented on a VIEWPixx monitor (VPIxx Technologies, Inc, Saint-Bruno, Quebec, Canada) at a viewing distance of 60 cm. The monitor had a spatial resolution of 1920 x 1080 pixel and a size of 51.5 x 29 cm. Eye movements of the right eye were recorded at 1000 Hz using a desktop mounted EyeLink 1000 (SR Research Ltd., Ontario, Canada) and the EyeLink Toolbox (53). Due to technical issues, the data of five participants were recorded at 2000 Hz and downsampled to 1000 Hz before further analysis.

### Materials

The stimuli of the experiment consisted of 36 digitized images of paintings taken from 12 different databases (Art Institute Chicago, Barnes Foundation, Brooklyn Museum, Lithuanian Art Museum, Mauritshuis, Metmuseum, Philadelphia Museum of Art, Rijksmuseum, The Athenaeum, Wikiart, Wikimedia Commons and Yale Center for British Art). The Athenaeum database is no longer available online. In order to prevent solving tasks based on merely image background information we chose lesser-known paintings with no or illegible signatures. We avoided paintings of human figures with highly salient face areas and incorporated texts. Images had a maximum resolution of 1300 x 800 pixels and were presented in front of a grey background. In order to sufficiently balance the stimuli, we narrowed down the art movement range of selected paintings to six art movements: Baroque, Expressionism, Impressionism, Cubism, Post-Impressionism, Romanticism and three media used: Oil, Pastel and Watercolor. Our stimuli set consisted of six paintings per six art movements. 12 of these paintings were oil paintings, 12—watercolor paintings and 12—pastel paintings. The first requirement for the successful stimuli-task assignment to each subject was that no participant could view the same painting twice. This way we could eliminate memory biases masking the task-depending viewing. The second requirement was that all paintings had to be shown under all three task conditions to ensure no differences occurred from image properties only. To fulfil these requirements, we first divided our 36 paintings in three sets with balanced style and medium categories. For each participant, each set was assigned with a different task. Images mapped to a certain task were then presented randomly in the experiment. Since we only had three sets and tasks, six participants would exhaust all, in this case, possible task-set combinations. To avoid the replication of task-set configurations a second arrangement of images to sets was introduced. We kept the balance of styles and media, but shuffled the positions of the paintings in the second arrangement. In the end, 12 participants per group could ensure the fulfilment of both requirements with 36 paintings rearranged in two different ways (see supplementary online material for more details). Information about date and medium of paintings was available from databases. Further, some databases (e.g. Wikiart) provided information about the style. We examined whether the provided information was credible by tracing the history timeline and representative artistic style information. Most of the time, given the date, technique used and subject matter of the painting, it was possible to define an art movement style. We used standard art movement definitions available in art history books (54).

To make sure that our operationalization of expertise was successful, we used an art expertise questionnaire from Pang et al. (23). Despite the lack of knowledge-related questions, according to Pang et al. (23), this measure covers the formal art education part of the expertise in greater detail compared to similar measures. It also contains questions about the amount of time spent on interaction with art and questions about formal art analysis skills. We analyzed 19 visual art-related questions with numerical information, the answer scores were summed up.

**Figure 1.**
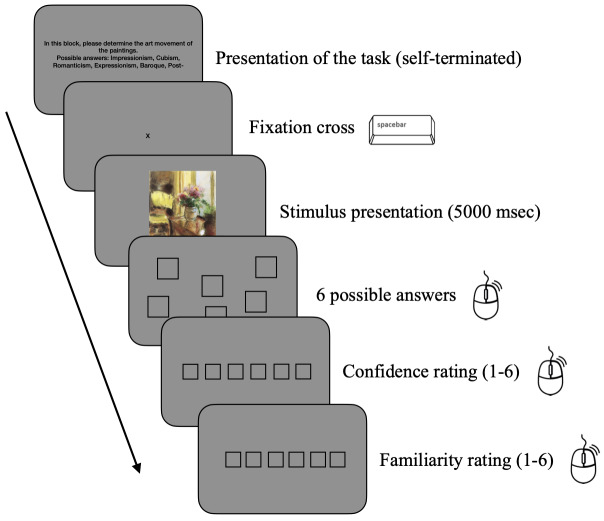
Trial Procedure. Each block started with a selfterminated task presentation followed by trials starting with a fixation cross. Trials consisted of a self-terminated fixation on a cross in the center of the screen, followed by the stimulus presentation for 5000 ms. After stimulus offset, the desired answer could be chosen from six spatially randomly presented answers with a mouse click. Subsequently confidence and familiarity ratings appeared successively on the screen and were entered using the mouse.

### Procedure

Participants were asked to fill in the consent form and answer the demographic questions. They were instructed about the experiment and tested for color deficiencies. The experiment consisted of three blocks, each containing 12 trials. The eye tracker was calibrated at the beginning of each block. Each block began with the presentation of the task and six possible answers. By fixating the fixation cross in the center of the screen and pressing the space bar participants started the presentation of the stimulus. Each painting was presented for 5000 milliseconds. The presentation could be terminated earlier by pressing the space bar, but only six participants (three experts, three non-experts) skipped the 5000 milliseconds viewing time. Participants could choose one correct answer from six possible answers with a mouse click. The six possible answers for the art movement task were Baroque, Expressionism, Impressionism, Cubism, Post-Impressionism, Romanticism. The six possible answers for the medium task were Oil, Pastel, Watercolor, Acrylic, Chalk and Ink. The six possible answers for the date task were chosen to roughly match the corresponding art periods: 1600-1730, 1904-1921, 1860-1910, 1912-1924, 1889-1935, 1762-1836. The answers were randomly presented on the screen to eliminate the position biases. Participants were also asked how confident they were in their answer on a scale of 1 to 6 (1very confident, 6-not confident) and how familiar was the painting on a scale of 1 to 6 (1-very familiar, 6-unfamiliar), ratings of familiarity and confidence were later reversed for the analysis (1-not confident, 6-very confident and 1-unfamiliar, 6-very familiar), so that the interpretation could be more intuitive. Participants could take a small break if needed before the calibration for the next block started. After the experiment they were asked to fill in the questionnaire.

### Design

The following independent variables were considered for the analysis: 1) expertise: experts (students of art related university courses) and non-experts in art (students of art unrelated university courses including social sciences and humanities), 2) task (style/art movement, date, medium). As dependent variables we considered: 1) accuracy of the task response, 2) familiarity rating of the painting, 3) confidence rating about the response, 4) fixation duration (ms), 5) saccade amplitude (deg).

### Analyses

We used the EyeLink 1000 algorithm with a combined velocity threshold of 30°/sec and acceleration threshold of 8000°/sec² to determine saccades. Fixations were detected if they fulfilled the criterion of not belonging to a saccade. We removed blinks and fixations outside the image. Fixations immediately preceding and following blinks were discarded. 120 saccades with unusual trajectories, long durations given their amplitudes (duration/amplitude>1.5) or notably large distances traveled by the eye given their amplitudes (distance/amplitude>3.5) were also excluded from the analysis. We used R (R Core Team, 2020) and lme4 package (55) to perform a linear mixed effects analysis (LME). We estimated four models with the dependent variables: accuracy, confidence, fixation duration and saccade amplitude. We entered the independent variables task and expertise as fixed effects, without interaction term into the models. As random effects, we entered intercepts for subjects and images. P-values were obtained by likelihood ratio tests of the full model with the effect in question against the model without the effect in question. The R package rmcorr (56) was used to calculate repeated measures correlation between familiarity and confidence. It accounts for non-independence by statistical adjustment of inter-individual variability.

### Data visualization

In order to provide an overview about the spatial distribution of eye movement parameters, we first visualized the scanpath per trial by plotting fixations and saccades on the corresponding paintings. To illustrate the fixation data, we aggregated fixation density maps, so-called heatmaps, for experts and non-experts, where all fixations for a single image divided by the sum of fixation durations were merged to form a density map. This fixation maps were smoothed by convolving a Gaussian kernel. They show the

accumulated time observers spent looking at the certain areas of the paintings by representing the values as colors. For 36 images, fixation densities across 13 experts and 14 non-experts for three tasks were aggregated into six maps. In order to facilitate the comparison additionally three difference density plots for both groups for each image were aggregated.

### Normalized Scanpath Saliency and Dispersion

Heatmaps showed that fixation distributions for the task medium had a slightly different appearance. After the detection of visually observable differences, we took a closer look and used the Normalized Scanpath Saliency (NSS) method, initially introduced by Peters et al. ( 57), to calculate the similarity of fixations across observers. Analogous to Dorr et al. (58), who used NSS method to evaluate the similarity of eye movements of multiple observers, we used a standard machine learning method “leave one out”. For each observer with a given task on a given image, the fixations of all other observers with the same task on the same image were used to create a fixation map. To this end, each fixation was modelled as a Gaussian with a standard deviation of 1.5° and all Gaussians were summed. This map was then normalized to have a mean of zero and a variance of unity. Finally, the fixation values at the fixations of the left-out observer were summed. Positive NSS indicates congruence between the regions fixated by one observer and all other observers. Zero values indicate uncorrelated fixations and negative NSS points out incoherent fixation locations. We also calculated dispersion – the size of spatial fixation distribution for experts and non-experts across all three tasks for all images as described in Holmqvist and Andersson (59). Each fixation was rated using a Gaussian with a standard deviation of 1.5°. For each trial all Gaussians were summed up and normalized to a maximum of 1. The values were then divided by the number of pixels so that the values could reside between 0 and 1.

## Results

### Behavioral parameters

**Figure 2.**
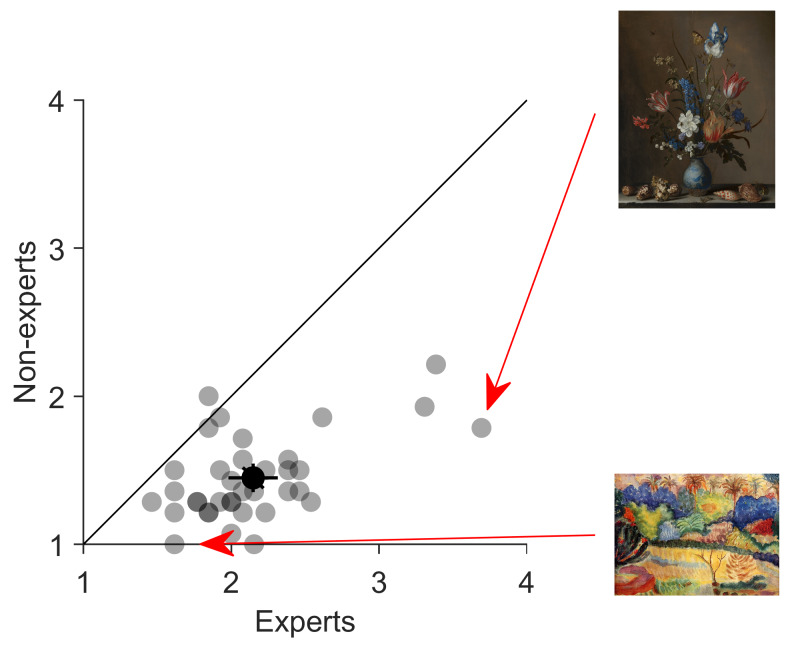
Average familiarity ratings of experts and nonexperts for 36 paintings. Half-transparent symbols indicate single images, the non-transparent symbol the mean across images. Error bars correspond to 95% confidence intervals. The paintings with the highest absolute familiarity value (painting by Balthasar van der Ast (29)) and with the lowest absolute familiarity value (painting by Paul Gauguin (6)) are shown on the right.

**Figure 3.**
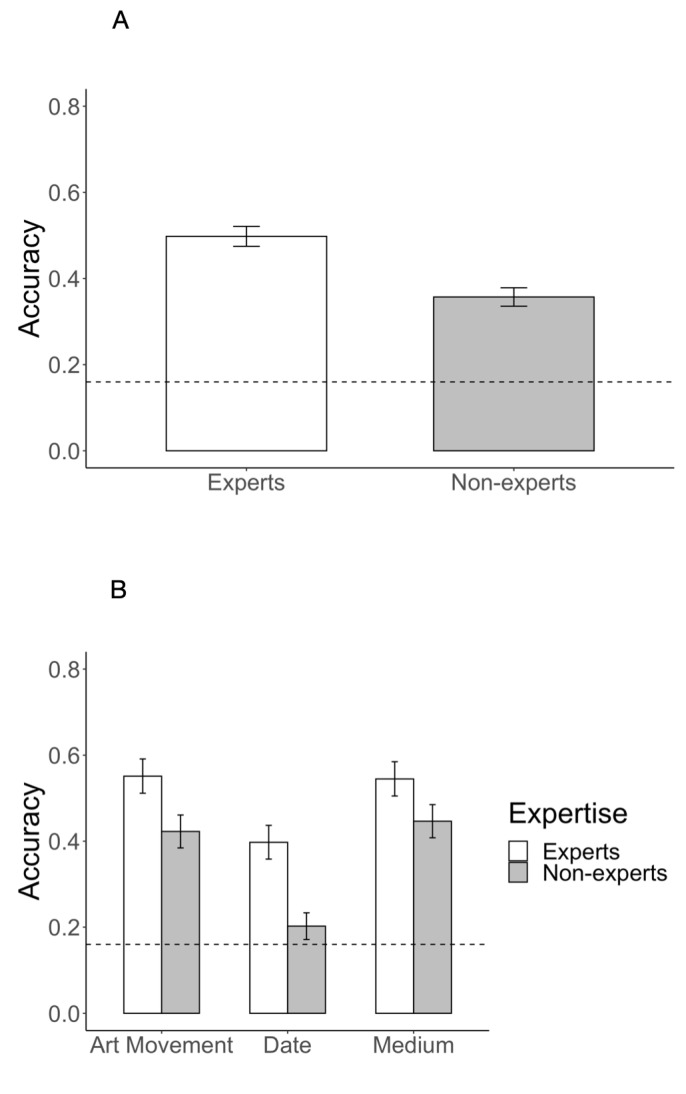
(A) Mean accuracy of experts and non-experts, (B) mean accuracy of experts and non-experts across all three tasks. Dashed line shows chance level of performance. Error bars correspond to 95% confidence intervals.

Experts achieved higher art expertise scores (*M* = 39.00, *SD* = 5.00) than non-experts (*M* = 22.00, *SD* = 6.30). The group difference was highly significant (*t*(24.49) = -7.82, *p* < 0.001). According to this result, our participant allocation to both groups might be regarded as reliable. We also found differences in the average task accuracy between two groups (Fig. 3, Table 1). Experts were more accurate in their answers than non-experts (*χ2*(1) = 9.31, *p* < 0.01). As chance performance would be at 0.16, both expert and non-expert performances were significantly above chance. Accuracy also varied between tasks (*χ2*(2) = 38.67, *p* < 0.001). Participants of this study had most difficulties with dating the paintings correctly ( *M* = 0.30, *SD* = 0.46) and were comparably accurate in the tasks – art movement (*M* = 0.48, *SD* = 0.50) and medium (*M* = 0.49, *SD* = 0.50). These differences in accuracy between groups and tasks were also reflected in the subjective confidence ratings. Reported confidence (Table 1) was lower by about 0.90 in non-experts compared to experts (*χ2*(1) = 8.51, *p* < 0.01). Subjective confidence varied between tasks (*χ2*(2) = 71.31, *p* < 0.001). Highest confidence was reported on the medium task (*M* = 4.19, *SD* = 1.46), followed by art movement ( *M* = 3.71, *SD* = 1.49) and date tasks ( *M* = 3.43, *SD* = 1.44). Reported confidence for correct answers (*M* = 4.31, *SD* = 1.40) was higher, than reported confidence for incorrect answers (*M* = 3.39, *SD* = 1.43). A correlational analysis revealed a weak correlation between confidence and familiarity (*r*(944) = 0.21, *p* < 0.001), indicating that subjects reported higher confidence on the more familiar paintings and lower confidence on the less familiar paintings. Overall, the reported familiarity of the paintings was quite low across all participants (*M* = 1.78, *SD* = 1.29). Our selection of paintings was even less familiar for non-experts than experts (*t*(35) = 10.105, *p* < 0.001). These results suggest the adequate selection of the stimuli in our experiment (Fig. 2).

**Table 1:** Mean values (M) and standard deviations (SD) of dependent variables, Normalized Scanpath Saliency (NSS) and Dispersion for both groups

	Experts		Non-experts	
	M	SD	M	SD
Expertise score (max. 70)	39.00	5.00	21.19	6.30
Accuracy	0.50	0.50	0.36	0.48
Confidence	4.25	1.36	3.34	1.48
Familiarity	2.15	1.45	1.45	1.02
Fixation duration (ms)	267.36	126.80	265.83	124.40
Saccade amplitude (°)	5.55	1.40	5.05	1.26
NSS	1.17	0.44	1.40	0.44
Dispersion	0.15	0.04	0.14	0.04

### Eye movement parameters

Fixations lasted on average 266.56 ms (*SD* = 125.55). The amplitude of saccades was on average 5.29° (*SD* = 3.80). These results are in line with previous work reporting an average saccade amplitude of 4°–6° on scenes that are on average 20°–30° wide ( 20, 60) and average fixation duration of 200–300 ms (61) in scene perception tasks. We could not find any effect of expertise on fixation duration (Table 1). In order to normalize the distribution, we subjected fixation duration to logarithmic transformation for the LME analysis. Whereas expertise did not have an effect on fixation duration, task influenced fixation duration (*χ2*(2) = 122.21, *p* < 0.001). The highest average fixation duration was detected during the medium task (*M* = 282.38, *SD* = 129.88), followed by the date task ( *M* = 260.76, *SD* = 123.76) and the art movement task (*M* = 257.37, *SD* = 121.70). These results suggest that participants’ fixations lasted longest when their task was to detect what medium was used for the painting. We found no significant interaction between expertise and task for fixation duration. Task also influenced saccade amplitude (*χ2*(2) = 13.87 *p* < 0.001) increasing it by about 0.05° for task date (*M* = 5.39, *SD* = 1.37) compared to art movement task ( *M* = 5.34, *SD* = 1.31) and reducing it by about 0.19° for task medium (*M* = 5.15, *SD* = 1.37) compared to art movement task. Consequently, in addition to longer fixation durations, task medium also induced smaller saccade amplitudes. This means that on average, our participants fixated longer and their eyes traveled a shorter distance during the medium task. We also found that in the medium task saccade amplitudes of non-experts were smaller (*M* = 4.83°, *SD* = 1.22), than experts’ (*M* = 5.49°, *SD* = 1.44), (Table 2). However, no significant interaction between expertise and task could be found using the LME model for saccade amplitude. We could also detect some differences between average saccade amplitudes on the group level (Table 1). Expertise had an effect on saccade amplitude (*χ2*(1) = 4.67, *p* < 0.05) reducing it by about 0.50° in non-experts compared to experts. From these results we can assume that expert group explored the paintings more extensively, as their eyes traveled larger distances across all three tasks.

**Table 2:** Mean values (M) and standard deviations (SD) of dependent variable, Normalized Scanpath Saliency (NSS) and Dispersion for three tasks split by Expertise

	Art movement				Date				Medium			
	Experts		Non-experts		Experts		Non-experts		Experts		Non-experts	
	Mean	SD	Mean	SD	Mean	SD	Mean	SD	Mean	SD	Mean	SD
Accuracy	0.55	0.50	0.42	0.50	0.40	0.49	0.20	0.40	0.55	0.50	0.45	0.50
Confidence	4.09	1.45	3.36	1.44	4.04	1.23	2.86	1.38	4.62	1.33	3.80	1.47
Fixation duration (ms)	260.83	40.47	265.42	52.14	269.26	39.92	263.49	48.97	288.79	48.61	288.50	50.49
Saccade amplitude (°)	5.55	1.36	5.15	1.24	5.62	1.39	5.17	1.31	5.49	1.44	4.83	1.22
NSS	1.37	0.42	1.58	0.39	1.28	0.42	1.54	0.40	0.86	0.31	1.08	0.35
Dispersion	0.15	0.04	0.13	0.03	0.15	0.04	0.14	0.04	0.16	0.04	0.15	0.04

### Normalized Scanpath Saliency and Dispersion

Variability in eye movement patterns was measured using the Normalized Scanpath Saliency method. We found rather high NSS values in our data (*max* = 2.69, *M* = 1.28) indicating an overall high coherence in fixation patterns between observers. Averaged NSS values for both groups are shown in Table 1, averaged NSS values for three tasks split by expertise are shown in Table 2. The LME model with expertise as fixed effect and image as random intercept revealed significantly higher average expert NSS score compared to average non-expert NSS score (*χ2*(1) = 43.89, *p* < 0.001) indicating larger variability of eye fixation distribution in experts. We also found the smallest mean NSS value for the task medium (*M* = 0.97, *SD* = 0.35) compared to two other tasks. The LME model with task as fixed effect and image as random effect suggested less coherence in eye movement patterns for the task medium ( *χ2*(2) = 131.35, *p* < 0.001), (Fig 5). The LME model with expertise as fixed effect and image as random intercept revealed higher dispersion values (*χ2*(1) = 21.42, *p* < 0.001) by experts (*M* = 0.15, *SD* = 0.04) compared to non-experts ( *M* = 0.14, *SD* = 0.04), indicating that they fixated larger areas of the paintings, compared to non-experts. The LME model with task as fixed effect and image as random intercept revealed that fixation dispersion significantly varied between tasks ( *χ2*(2) = 43.47, *p* < 0.001). The highest spatial fixation dispersion value was found for the medium detection task (*M* = 0.16, *SD* = 0.04), compared to art movement (*M* = 0.14, *SD* = 0.04) and date (*M* = 0.14, *SD* = 0.04) tasks, suggesting that both experts and non-experts fixated larger areas of the paintings during the medium detection task. The dependent values for all three tasks split by expertise can be found in Table 2.

### Temporal differences

To investigate temporal information, we plotted fixation durations and saccade amplitudes over the course of a trial. Previous research has shown that the first fixation contains a strong central bias (62) and differs from later fixations (63). The average duration of the initial fixation in our data (*M* = 384.97, *SD* = 130.63) was longer than the overall average fixation duration ( *M* = 266.56, *SD* = 125.55). The average amplitude of the initial saccade (*M* = 3.19°, *SD* = 1.89) was smaller than the average saccade amplitude (*M* = 5.29°, *SD* = 1.35). Similar results were reported by Over et al. (63) and Van Loon et al. ( 64). We therefore excluded the first fixations and first saccades from the analysis and investigated fixations and saccades in the ordinal number range of 2–13. As expected, we found increasing fixation durations as a function of ordinal fixation number. The LME model with fixation number as fixed effect and subject and image as random effects revealed a significant effect of ordinal fixation number on fixation duration (*χ2*(11) = 370.37, *p* < 0.001).

We also observed amplitudes becoming shorter across the trial. The LME model with saccade number as fixed effect and subject and image as random effects revealed a significant effect of ordinal saccade number on saccade amplitude (*χ2*(11) = 69.00, *p* < 0.001). Fixation durations and saccade amplitudes for each task plotted by ordinal fixation and ordinal saccade number and fixation durations and saccade amplitudes for experts and non-experts plotted by ordinal fixation and saccade number are shown in Figure 6.

## Discussion

**Figure 4.**
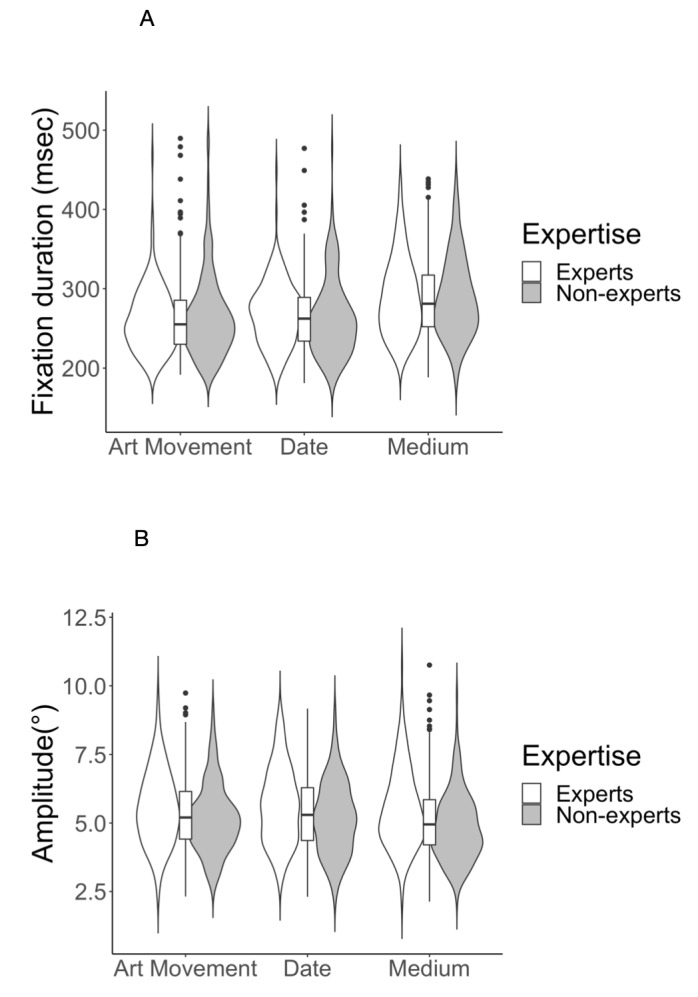
(A) Average fixation duration and (B) average saccade amplitude for experts and non-experts across three tasks.

**Figure 5.**
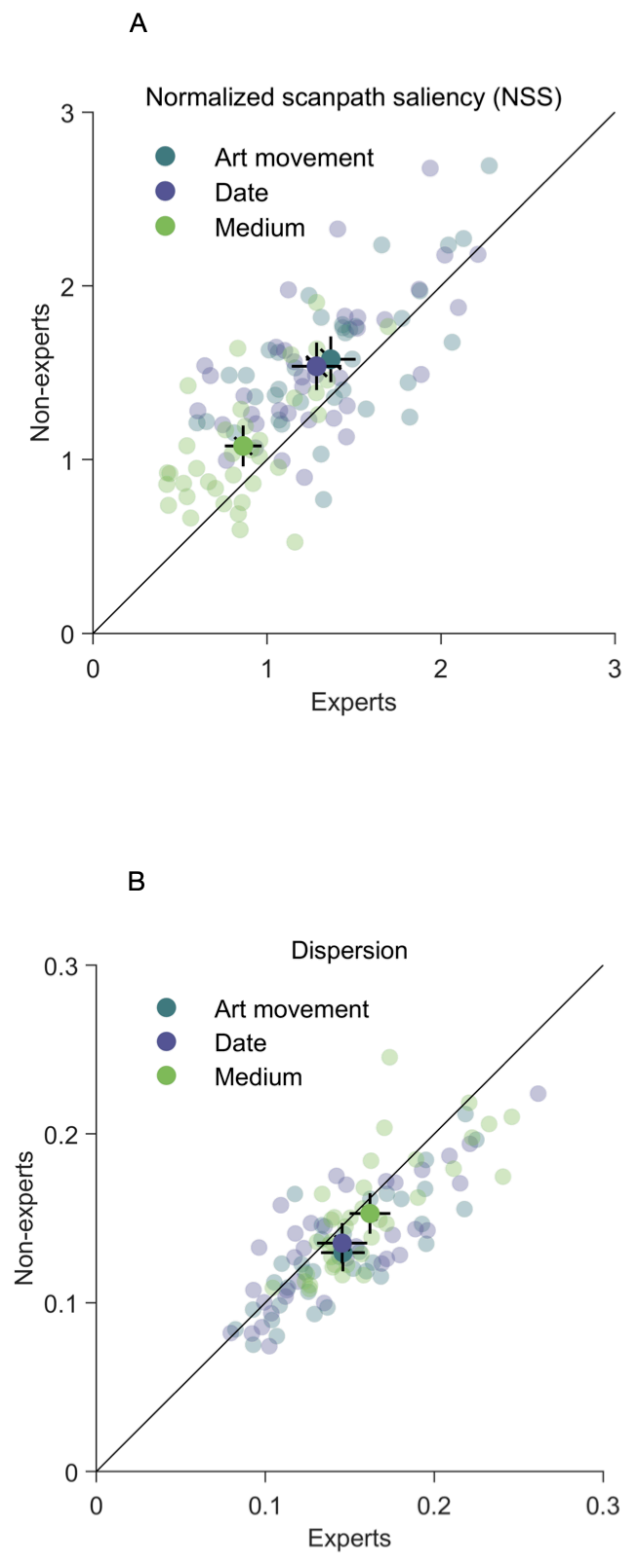
Average NSS (A) and Dispersion (B) values, within groups, within tasks comparison. Half-transparent symbols indicate single images, the non-transparent symbols the mean across images. Error bars correspond to 95% confidence intervals.

In this study, we examined the eye movements of observers with art education and observers without art education background, while they were viewing 36 digitized paintings under three task conditions. Participants were asked to identify the style/art movement, date or medium of the paintings. We first looked at the behavioral parameters. The questionnaire results reassured the optimal allocation of participants to the groups (Table 1). Accuracy test showed that experts, although not being highly accurate, outperformed non-experts and chance level of accuracy (Fig. 3). Experts’ superior performance in style/art movement detection supports Augustin and Leder’s (42) finding that experts are better at style processing and is in line with Leder et al.’s (41) model prediction. Dating of the paintings was the most difficult task for all participants, particularly for novices (Fig. 3(B)). Familiarity levels were low and comparable for all images. Our stimulus selection can be therefore regarded as adequate (Fig. 2). Participants were more confident in their answers about the paintings they were more familiar with. In general, experts were more confident (Table 1). Confidence was also higher for correct answers and highest for the medium detection task. In order to explore task-dependent viewing strategies of our participants, we examined the eye movement parameters including fixation durations and saccade amplitudes. As expected, we found task-induced differences in fixation durations and saccade amplitudes. Longest fixation durations were found in the medium detection trials (Fig. 4(A)). In other words, detecting what paint was used for the painting, whether it was oil, watercolor, pastel, etc. resulted in longer fixations compared to the two other tasks. In addition to longer fixations, the medium task induced shorter saccade amplitudes, however non-experts decreased their saccade amplitudes to a slightly larger extent, compared to experts during the same task (Fig. 4(B)). We also examined the similarity of fixation patterns of experts and non-experts in the different tasks. Our results showed overall less coherence and higher dispersion in fixation locations of experts compared to non-experts, indicating greater image coverage by experts. We also found less coherence and higher dispersion in fixation locations for the task medium (Fig. 5). This indicates that while participants tried to detect medium of the paintings, the distributions of fixations they made were more diverse compared to two other tasks. This difference regarding medium task can be explained by the assumption, that informative areas for the medium task were more evenly distributed across the painting and allowed spatially more diverse fixations. Altogether, our findings imply that observers adjusted how they searched for information in the paintings depending on the task.

Generally, experts showed larger amplitudes across all three tasks (Table 1) and fixated larger areas of the paintings. These findings are in line with previous studies supporting more holistic scanning in art experts (28, 47) and are compatible with the results reported by Pihko et al. (27) who observed larger distance of fixations from the image center in experts.

**Figure 6.**
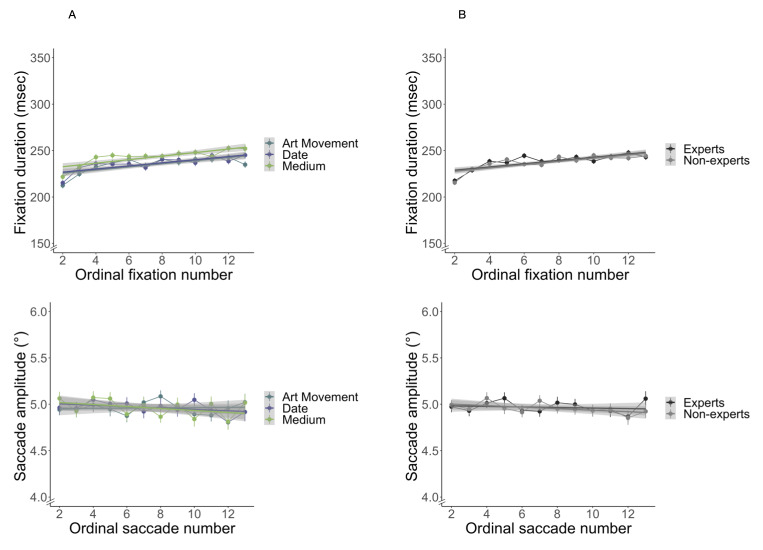
Intergroup fixation density correlations for three tasks, (B) correlations between tasks for experts and non-experts

Temporal analysis of the trial showed the pattern of fixations becoming longer and saccades becoming smaller over the trial, regardless of task or expertise (Fig. 6). These results are in line with a well-established observation that fixation durations gradually increase after stimulus onset, while saccade amplitudes decrease (10, 60, 65).

How our overall findings can be interpreted within a broader context of expertise guided visual search? If we think about theories from Gegenfurtner et al.’s (31) meta-analysis, our result of larger saccade amplitudes in the expert group supports the first assumption of Kundel et al.’s (35) holistic model of image perception. The second assumption of the model is that experts are also first to fixate task-relevant regions. This assumption could not be tested in this study.

According to the model, experts are able to rapidly extract a global scene impression during the initial scan and compare this impression with the prior knowledge about the image category. After this processing, experts need less time to first fixate task-relevant area. The model assumes that during the initial global scan, experts, due to their visual experience, are better at extracting information from their parafoveal and peripheral vision. The initial ambient mode of processing is then replaced with the focal mode of processing, where information is examined in more detail (66).

We know that visual expertise is domain specific and is based on a very specific knowledge of concrete image category. There is a huge amount of mixed results on expert fixation durations and saccade amplitude sizes depending on the visual domain (31, 32). However, generalizations about expertise within a domain should also be made with caution, as we have seen from our results, eye movement parameters very much depend on stimuli and difficulty of the task. This variability in stimuli and tasks might be the reason for the mixed results regarding fixations and amplitudes across the art domain. This work was the first attempt to examine task influences on art viewing. Future research should shed more light on what stimuli and task characteristics constitute to the differences across visual domains.

### Limitations

Our work clearly has some limitations. The stimuli set used in this study consisted of only 36 paintings covering only six art periods. A relatively low number of paintings, compared to other studies, for example, Wallraven et al. (67) who selected 550 paintings covering 11 art periods for their style categorization study. Additionally, our artist coverage was biased. The stimulus set consisted of 36 paintings by 30 painters, with four paintings belonging to one painter. Although, the low familiarity rates and superior expert accuracy validates our choice of paintings, we believe that the balancing of stimulus set features, always has room for improvement. For example, due to the fact that we only used six art periods, the distribution of the art periods across art history timeline was not perfect. Skipping art periods between Baroque and Romanticism and periods between Romanticism and Impressionism led to gaps between years in the answer options. Conceivably, this could facilitate discrimination of some art movements compared to others according to the answer options.

Another controversial issue is whether students of art university departments fulfil the criteria of art expertise, the problem also discussed in Bauer and Schwan (48). According to Ericsson and Lehmann ( 30), experts in most domains attain their highest performance levels after a decade of intensive training. Although, the level of art expertise in our expert group was clearly higher than in the control group, nearly half of our experts were bachelor students in their second or third year of study, corresponding at most to an intermediate level of expertise. We assume that if we had an opportunity to test well-experienced art experts with prolonged training history, we could find even more profound differences between two groups.

Another limitation of our study, often discussed in the laboratory studies of art (27), is that digitized paintings obviously do not have the same qualities as the original paintings. Whether it is sufficient to use digitized copies of paintings usually depends on the question of the study. For instance, it can be problematic to use digitized copies when measuring affective responses to paintings (68, 69). In our case, since conservator-restorers and art experts usually work with the original paintings while solving the same tasks we gave our participants, we probably deprived our subjects of having some additional textural cues. Different physical-reflection properties on the surface of original paintings might be helpful for low-level feature extraction from the paintings.

It has been shown that liking and aesthetic preference is associated with longer fixation durations (70), probably also for non-biological object categories relevant for our sample of stimuli. However, since paintings were equally often assigned to three tasks and two groups, aesthetic differences between paintings can be disregarded. In order to explore the interaction of aesthetic preferences and task demands it would be interesting to include aesthetic preference ratings in future task-dependent art research.

As already discussed, task-induced differences highly depend on the variability between the distribution of informative locations in our images. The reason why we could not find any differences between the tasks date and art movement might be that informative areas of these tasks were overlapping. Although, observers were less accurate in the dating task, it can be assumed that two tasks are interconnected, considering the fact that if one can recognize the style, approximate date can be estimated and vice versa. It is conceivable that by setting these two tasks we simply asked the same question differently. The difficulty of predefining informative areas of the paintings for our three tasks without further inspection can be regarded as a general limitation of art stimuli. There are no general rules in visual art that can guide the gaze of observer through consecutive relevant areas, similar to mnemonic guides in medicine (e.g. **A**irways, **B**ones, **C**ardiac silhouette, **D**iaphragm) that help routinizing the eye movements of physicians.

### Outlook

Our findings are consistent with the general assumption that observers adjust their viewing behavior according to the task. Overall, our results lend support to the notion, assuming experts’ superiority in extracting task-relevant information based on their knowledge of scene content and scene statistics. Further investigation is needed to determine whether experts in art are indeed better in extracting task-relevant information than novices. For this purpose, criteria for informativeness needs to be proposed. Relevant ROIs in each painting and for each task can be defined according to the criteria. This way, the second assumption of the holistic model of image processing can also be tested. It would be interesting to determine whether art experts, similar to medical experts (32) are faster in fixating task-relevant information. Future research might apply the meaning maps approach (71) to examine how semantic meaning distribution varies as a function of a task and what is the role of semantic representations in attention guidance in art viewing.

Even though viewing of art undoubtedly underlies human plans and task demands, this study is only the first step towards enhancing our understanding of task influences during art viewing. In addition to improving our understanding of task and expertise effects on eye movements, studies on art can give us some insights about art perception, sometimes with practical implications. For example, expertise-novice comparison can provide valuable information for the question of how to teach visual literacy, i.e. the ability to find meaning in imagery and efficient viewing strategies at art schools. Eye movement analysis generally, provides limitless possibilities of examining art history theories, artistic idiosyncrasies, exhibition concepts and many more. Findings from eye-tracking studies can lead to new ideas for enhancing visitor experiences in museums. As the quality of mobile eye-tracking improves, it certainly will accelerate research in the museum context, increasing ecological validity of the studies (72–74).

### Conclusion

To sum up, we were able to show that, as in other known domains, task clearly influences eye movements during art viewing. We found task-induced differences in fixation durations and saccade amplitudes. Longest fixations and shortest saccade amplitudes were observed when art viewers were looking for cues to detect what paint material was used in the painting. These results can be interpreted in favor of observers’ implemented task-specific strategy. It can be assumed that effortlessly detectable informative areas, in case of medium detection task, induced greater fixation variability and higher dispersion among observers, facilitated focused processing and ensured satisfactory task accuracy. With regard to expertise, we found less coherent fixation patterns among experts, suggesting more diversity in task-solving in this group. Our data also illustrates the effect of expertise on average saccade amplitude, supporting expertise-related holistic processing theory.

## Ethics and Conflict of Interest

The author(s) declare(s) that the contents of the article are in agreement with the ethics described in http://biblio.unibe.ch/portale/elibrary/BOP/jemr/ethics.html and that there is no conflict of interest regarding the publication of this paper.

## Acknowledgements

This work was supported by the Deutsche Forschungsgemeinschaft (DFG, German Research Foundation) project number 222641018 – SFB/TRR 135 TP B2. Data and stimuli are available at: http://doi.org/10.5281/zenodo.3956829.
